# Diabetic microvascular complications are associated with reduced global longitudinal strain independent of atherosclerotic coronary artery disease in asymptomatic patients with diabetes mellitus: a cross-sectional study

**DOI:** 10.1186/s12872-021-02063-w

**Published:** 2021-06-02

**Authors:** Gokulan Pararajasingam, Laurits Juhl Heinsen, Johanna Larsson, Thomas Rueskov Andersen, Brian Bridal Løgstrup, Søren Auscher, Jørgen Hangaard, Rasmus Møgelvang, Kenneth Egstrup

**Affiliations:** 1 Cardiovascular Research Unit, Odense University Hospital Svendborg, Baagøes Allé 15, 5700 Svendborg, Denmark; 2 Department of Cardiology, Aarhus University Hospital Skejby, Palle Juul Jensens Boulevard 99, 8200 Aarhus, Denmark; 3 Department of Internal Medicine (Cardiology), Odense University Hospital Svendborg, Baagøes Allé 15, 5700 Svendborg, Denmark; 4 Department of Internal Medicine (Endocrinology), Odense University Hospital Svendborg, Baagøes Allé 15, 5700 Svendborg, Denmark; 5 Heart Centre, Copenhagen University Hospital Rigshospitalet, Blegdamsvej 9, 2100 Copenhagen, Denmark

**Keywords:** Diabetes mellitus, Global longitudinal strain, Echocardiography, Plaque burden, Asymptomatic, Microvascular complications

## Abstract

**Background:**

Reduced left ventricular function, assessed by global longitudinal strain (GLS), is sometimes observed in asymptomatic patients with diabetes mellitus (DM) and is often present in patients with diabetes-related microvascular complications. Our aim was to assess the association between microvascular complications, coronary artery plaque burden (PB) and GLS in asymptomatic patients with DM and non-obstructive coronary artery disease (CAD).

**Methods:**

This cross-sectional study included patients with DM without any history, symptoms or objective evidence of obstructive CAD. All patients were identified in the outpatient Clinic of Endocrinology at Odense University Hospital Svendborg. An echocardiography and a coronary computed tomography angiography were performed to assess GLS and the degree of CAD, respectively. A coronary artery stenosis < 50% was considered non-obstructive. A linear regression model was used to evaluate the impact of potential confounders on GLS with adjustment of body mass index (BMI), mean arterial pressure (MAP), microvascular complications, type of diabetes, tissue Doppler average early diastolic mitral annulus velocity (e’) and PB.

**Results:**

Two hundred and twenty-two patients were included, of whom 172 (77%) had type 2 DM and 50 (23%) had type 1 diabetes. One hundred and eleven (50%) patients had microvascular complications. GLS decreased as the burden of microvascular complications increased (P-trend = 0.01): no microvascular complications, GLS (− 16.4 ± 2.5%), 1 microvascular complication (− 16.0 ± 2.5%) and 2–3 microvascular complications (− 14.9 ± 2.8%). The reduction in GLS remained significant after multivariable adjustment (β 0.50 [95% CI 0.11–0.88], *p* = 0.01). BMI (β 0.12 [95% CI 0.05–0.19]) and MAP (β 0.05 [95% CI 0.01–0.08]) were associated with reduced GLS. In addition, an increased number of microvascular complications was associated with increased PB (β 2.97 [95% CI 0.42–5.51], *p* = 0.02) in a univariable linear regression model, whereas there was no significant association between PB and GLS.

**Conclusions:**

The burden of microvascular complications was associated with reduced GLS independent of other cardiovascular risk factors in asymptomatic patients with DM and non-obstructive CAD. In addition, the burden of microvascular complications was associated with increasing PB, whereas PB was not associated with GLS.

**Supplementary Information:**

The online version contains supplementary material available at 10.1186/s12872-021-02063-w.

## Background

Patients with type 2 diabetes (T2DM) are at increased risk of coronary artery disease (CAD), which is the leading cause of morbidity and mortality among these patients [1–3]. Furthermore, diabetes mellitus (DM) has been associated with the development of microvascular complications such as albuminuria, retinopathy and peripheral neuropathy [4, 5]. The detection of CAD remains challenging in patients with DM due to the atypical presentation of symptoms, but also due to the presence of silent ischaemia [6, 7]. Several studies have investigated changes in left ventricular (LV) systolic function in asymptomatic patients with T2DM by using longitudinal strain [8–10]. However, a limitation of these studies was the use of indirect testing for significant CAD, including exercise testing, stress echocardiography and single-photon emission computed tomography (SPECT). Impairment of LV systolic function could be explained by the presence of atherosclerosis, but studies have also indicated a coronary microvascular component associated with diabetes-related microvascular complications [11, 12]. Additionally, it has been demonstrated that patients with DM have decreased coronary flow reserve (CFR) as a marker of microvascular dysfunction in the myocardium in patients with acute myocardial infarction [13] and patients with diabetic retinopathy [14].

To our knowledge, no studies have yet investigated the interplay between LV systolic function and the presence of diabetes-related microvascular complications in asymptomatic DM patients with confirmed non-obstructive CAD assessed by coronary computed tomography angiography (CCTA). We aimed to assess the association between diabetes-related microvascular complications, global longitudinal strain (GLS) and coronary artery plaque burden (PB) in asymptomatic patients with DM and non-obstructive CAD (< 50%).

## Methods

### Study population

This cross-sectional, single-centre study included patients with DM identified in the outpatient Clinic of Endocrinology at Odense University Hospital Svendborg. Patients were enrolled between March 2016 and August 2017 and were examined with 2D transthoracic echocardiography (TTE), CCTA, electrocardiography (ECG), blood pressure measurements, medical review and blood samples. The inclusion criteria were as follows: age ≥ 18 years, ability to give informed consent, an estimated glomerular filtration rate (eGFR) ≥ 45 ml/min/m^2^ and a verified diagnosis of DM using international standards [15]. The exclusion criteria were as follows: Dyspnea corresponding to New York Heart Association classification III/IV, symptoms suggestive of CAD, left ventricular ejection fraction (LVEF) < 40%, atrial fibrillation, moderate-to-severe aortic/mitral valve stenosis or insufficiency, poor echocardiographic acoustic window, allergy to iodinated contrast agent, coronary artery stenosis (≥ 50%) as shown by CCTA, chronic obstructive pulmonary disease (COPD) and use of inhalation medication, bronchial asthma, a history of myocardial infarction, percutaneous coronary intervention and coronary artery bypass graft (CABG).

### Clinical data

Data on age, gender, height and weight were collected and body mass index (BMI) was calculated. Patients were registered as having a medical history of hypertension through a combination of patient reports and regular use of antihypertensive medication. Hyperlipidaemia was defined as any regular prescription for cholesterol-lowering medication such as statins, selective cholesterol absorption inhibitors and/or fibrates. Familial predisposition to ischaemic heart disease (IHD) was defined as cardiovascular disease in a first-degree relative with clinical presentation before the age of 55 in male relatives and the age of 65 in female relatives. Smoking status was categorized as active smokers, previous smokers and non-smokers. Exposure to smoke was calculated in pack-years, which was the number of years with a daily consumption of 20 cigarettes. Heart rate (HR), systolic blood pressure (SBP) and diastolic blood pressure (DBP) were measured twice after a rest period. Data on DM duration were obtained from the Funen diabetes database (FDDB) [16].

### Diabetes-related microvascular complications

Diabetes-related microvascular complications (albuminuria, retinopathy and peripheral neuropathy) were obtained from the FDDB. Albuminuria was considered a surrogate of nephropathy and was based on the urine albumin-to-creatinine ratio (UACR). Patients were categorized as having normoalbuminuria (UACR < 30 mg/g), microalbuminuria (30 mg/g ≥ UACR < 300 mg/g) or macroalbuminuria (UACR ≥ 300 mg/g). Chronic kidney disease stage (CKD) was categorized as follows: No CKD (eGFR > 90 ml/min/m^2^), mild CKD (60–89 ml/min/m^2^) or moderate CKD stage IIIa (45–59 ml/min/m^2^). Peripheral neuropathy was categorized as normal sensitivity, reduced sensitivity or no sensitivity by biothesiometry. Retinopathy was categorized as no retinopathy, mild non-proliferative diabetic retinopathy (NPDR), moderate NPDR, severe NPDR, proliferative diabetic retinopathy and/or clinically significant macular oedema. The number of microvascular complications was counted and ranged between 0 and 3 microvascular complications.

### Biochemistry

Blood samples were collected from each patient and assessed for biochemical parameters such as haemoglobin, creatinine, high sensitivity troponin T (TnT), glycated haemoglobin A1c (HbA1c), low-density lipoprotein (LDL) cholesterol, high-density lipoprotein (HDL) cholesterol, total cholesterol and triglyceride levels and eGFR adjusted by body surface area (BSA).

### Medication

All patients underwent a medical review. The types of medication, dosages and duration of antidiabetic and cardiovascular medications were recorded in this study. Specifically: Acetylsalicylic acid (ASA), clopidogrel, angiotensin-converting enzyme (ACE), aldosterone receptor blocker (ARB), calcium antagonists, beta blockers, diuretics (loop, thiazide and mineralocorticoid receptor antagonists), biguanides, sodium-glucose cotransporter-2 (SGLT-2) inhibitors, dipeptidyl peptidase 4 (DPP-4) inhibitors, glucagon-like peptide-1 (GLP-1) agonists and insulin.

### Echocardiography

TTE was performed using a Vivid E90 and Vivid E95 (GE Healthcare), and all images were pseudonymized and stored for offline analysis. All measurements were performed by one observer (GP). Conventional 2D measurements were obtained and included indexed left atrial volume (LAVI) by Simpson’s biplane method, LVEF by Simpson’s biplane method and dimensions of the left ventricle. Left ventricle mass (LVM) was calculated based on the modified Devereux formula (linear method): 0.8 × 1.04 x (left ventricle end-diastolic dimension + posterior wall end-diastolic dimension + interventricular septum end-diastolic dimension) ^ 3 − (left ventricle end-diastolic dimension) ^ 3) + 0.6 g. LVM was indexed (LVMI) by BSA using the Dubois formula (0.00718 × (height [cm] ^ 0.725) × (weight [kg] ^ 0.425). Pulsed-wave (PW) Doppler measurements of the transmitral early peak velocity (E), transmitral peak late velocity (A) and deceleration of transmitral early peak velocity (DCT) were obtained. Lateral and septal PW tissue Doppler was placed to calculate the average early diastolic mitral annulus velocity (e’). Left ventricular filling pressure was calculated as (E/e’). The isovolumetric relaxation time (IVRT) was calculated as the average lateral and septal values of the duration from closure of the aortic valve to opening of the mitral valve.

### Global longitudinal strain

Peak systolic longitudinal strain was assessed using automated function imaging in EchoPAC software version 202, revision 50 (GE Healthcare). These measurements were obtained from 2D greyscale images of the apical four-chamber, two-chamber and long-axis views with an optimized frame rate of 60–80 frames/s. Three points were anchored inside the myocardial tissue, two were placed at the basal segments along the mitral valve annulus and one at the apex. An automatic algorithm was initiated to suggest a region of interest (ROI) within the myocardial tissue and the movement of the ROI. Visual inspection of both ROIs and movement during a cardiac cycle was performed and approved, if applicable. Automatic aortic valve closure (AVC) timing was suggested and adjusted manually after visual inspection.

### Coronary computed tomography angiography

Patients were prepared for CCTA with a sinus node inhibitor (Ivabradine, 7.5 mg) prior to the scan. Patients with HR > 65 beats/min following Ivabradine ingestion were given a beta blocker. The CCTA and the echocardiography were placed on separate days, when possible. Sublingual fast-acting nitrate was administered just before CCTA. Images were obtained on a Revolution CT (GE Healthcare) scanner with an ECG-gated prospective acquisition in 75% of the R–R interval with an additional padding of 45 ms. Some patients had an additional phase acquired in the 40% phase of the R–R interval in case of increased HR. A fixed volume of 60 ml of iodine contrast (Visipaque, 320 mg iodine/ml) was administered and the scan was timed visually by the maximal contrast attenuation in the ascending aorta. Tube voltage and current were modulated to the body size of the patients and ranged between 80–140 kV and 150–700 milliamps, respectively. All available phases were reconstructed and analysed offline with a semiautomatic software (Qangio CT Research Edition version 3.1.3.18, Medis Netherlands). The major epicardial vessels, the left main artery (LM), left anterior descending artery (LAD), circumflex artery (CX) and right coronary artery (RCA), were screened for the presence of plaque (segments 1, 2, 3, 5, 6, 7, 8, 11, 13, and 15) according to the American Heart Association 17-segment model [17]. Segments with plaque underwent semiautomatic plaque analysis. All measurements of plaque volumes were performed by 1 observer (LJH) blinded to all clinical data. Total plaque volume (TPV) was calculated as (total vessel volume − total lumen volume). The plaque burden (PB) was calculated as the ratio of TPV to total vessel volume. Furthermore, coronary artery calcium (CAC) was acquired for all patients prior to the contrast-enhanced scan. A coronary artery stenosis (≥ 50%) by CCTA was considered obstructive.

### Statistical analysis

Continuous variables were expressed as the mean and standard deviation (SD). The normality of the distribution was assessed by quantile–quantile plots. Variables with non-Gaussian distributions were expressed as medians and corresponding interquartile range (IQR). Categorical variables were expressed as numbers and percentages. Kruskal–Wallis one-way analysis of variance, Student’s *t* test and Pearson’s chi-squared test were used to test for differences between groups. Cuzick’s test was used to test for trends between groups. Beta coefficients (β) and corresponding 95% confidence intervals (95% CI) were provided for univariable and multivariable linear regression models. All statistical tests were two-sided and a *p*-value < 0.05 was considered statistically significant.

#### Sample size

This cross-sectional study was a sub study from a larger clinical study with a longitudinal study design (not yet published). There was no pre-hoc calculation of sample size for this particular sub study, but previous studies have included similar number of subjects with DM [18–20]. The multivariable linear regression models complied with the statistical considerations regarding the minimum number of subjects per variables in order to estimate regression coefficients adequately [21].

#### Linear regression model

The associations of GLS with relevant confounders were initially assessed in a univariable regression model. Potential confounders were defined as age, gender, blood pressure, BMI, microvascular complications (albuminuria, neuropathy and retinopathy), type of diabetes, diabetes duration, HbA1c level and PB. Medication and echocardiographic parameters were included in the regression model, only when there were any significant differences between groups. Significant confounders in the univariable regression model were then tested in a multivariable regression model. Exploratory regression models were used to evaluate the association between the subgroups of microvascular complications and GLS, but also to evaluate the association between the number of microvascular complications and PB.

#### Reproducibility

Intra-observer and inter-observer reproducibility of GLS and PB were assessed using 20 patients and evaluated with the mean difference and a corresponding limit of agreement (LOA). GLS and PB were assessed by 2 observers (GP/TRA) blinded to the clinical data.

## Results

**Fig. 1:**
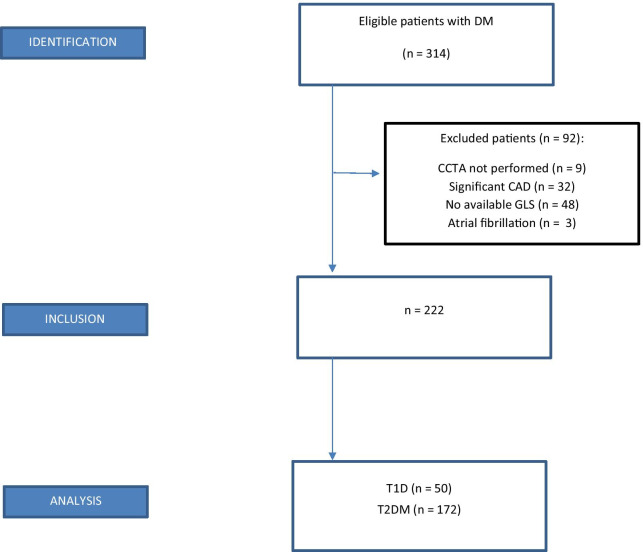
STROBE flowchart. *STROBE* Strengthening the Reporting of Observational Studies in Epidemiology; *CCTA* coronary computed tomography angiography; *CAD* coronary artery disease; *GLS* global longitudinal strain; *T1D* type 1 diabetes; *T2DM* type 2 diabetes mellitus

**Fig. 2:**
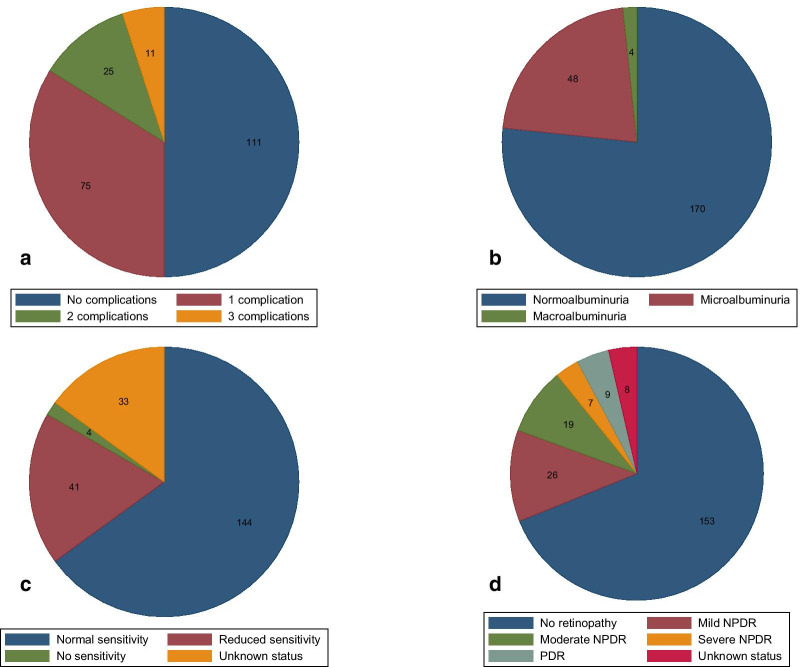
Burden of microvascular complications and subgroups of microvascular complications in 222 patients. Pie charts with an overview of microvascular complications. A) Distribution of patients with a number of microvascular complications ranging from 0 to 3. B) Distribution of patients with albuminuria. C) Distribution of patients with peripheral neuropathy. D) Distribution of patients with diabetic retinopathy. *NPDR* non-proliferative diabetic retinopathy; *PDR* proliferative diabetic retinopathy

This study included 222 patients, of whom 172 (77%) had T2DM (Fig. 1). All baseline characteristics are displayed in Table 1. The mean age was 59 years with a range from 35 to 78 years. One hundred and fifty-one (68%) patients were men, 137 (62%) were being treated for hypertension, and 159 (72%) were being treated for hyperlipidemia. The mean duration of diabetes was 13 years with a range from 0 to 54 years. One hundred and eleven (50%) patients had at least one diabetes-related microvascular complication (albuminuria, retinopathy or neuropathy) (Fig. 2). Of these, 75 (68%) patients had one microvascular complication, 25 (22%) patients had two microvascular complications and 11 (10%) patients had three microvascular complications (Table 2).

**Table 1:** Characteristics of clinical data, biochemistry and medication stratified by microvascular complications

	No complications (n = 111)	Any complications (n = 111)	*p*
Clinical			
Age (years)	58 ± 9	59 ± 10	ns
Men, n (%)	67 (60)	84 (76)	0.01
Height (cm)	174 ± 9	176 ± 9	ns
Weight (kg)	90 ± 17	93 ± 17	ns
BMI (kg/m^2^)	30 ± 5	30 ± 5	ns
Hypertension, n (%)	64 (58)	73 (66)	ns
Hyperlipidaemia, n (%)	77 (69)	82 (74)	ns
Familial predisposition to IHD, n (%)	16 (14)	13 (12)	ns
Smoking exposure (pack-years) [IQR]	2 [0–25]	9 [0–29]	ns *
MAP (mmHg)	103 ± 10	104 ± 10	ns
HR (beats/minute)	67 ± 13	71 ± 12	0.03
Diabetes duration (years)	11 ± 9	16 ± 11	< 0.001
T2DM, n (%)	88 (79)	84 (76)	ns
Biochemistry			
Haemoglobin (mmol/L)	8.6 ± 0.7	8.5 ± 0.9	ns
eGFR (ml/min/m^2^)	75 ± 9	72 ± 12	0.05
HbA1c (mmol/mol)	59 ± 13	63 ± 15	0.02
LDL (mmol/L)	2.0 ± 0.8	2.2 ± 0.9	ns
HDL (mmol/L)	1.3 ± 0.5	1.3 ± 0.4	ns
Total cholesterol (mmol/L)	4.1 ± 1.0	4.2 ± 1.1	ns
Triglycerides (mmol/L) [IQR]	1.5 [1.1–2.4]	1.6 [1.0–2.5]	ns*
Troponin T (ng/L) [IQR]	6 [4–9]	8 [5–12]	0.01*
UACR (mg/g) [IQR]	9 [5–13]	24 [8–69]	< 0.001*
Medication			
Acetylsalicylic acid, n (%)	6 (5)	16 (14)	0.03
Clopidogrel, n (%)	2 (2)	6 (5)	ns
Beta blockers, n (%)	12 (11)	10 (9)	ns
Statins, n (%)	69 (62)	77 (69)	ns
ACE inhibitor/ARB, n (%)	59 (53)	69 (62)	ns
Calcium blockers, n (%)	19 (17)	33 (29)	0.03
Diuretics, n (%)	31 (28)	37 (33)	ns
Biguanides, n (%)	73 (66)	70 (63)	ns
SGLT-2 inhibitor, n (%)	8 (7)	9 (8)	ns
DDP-4 inhibitor, n (%)	16 (14)	16 (14)	ns
Sulfonylureas, n (%)	13 (12)	19 (17)	ns
GLP-1 receptor agonists, n (%)	17 (15)	22 (20)	ns
Insulin, n (%)	52 (47)	64 (58)	ns

*ns* not significant; *BMI* body mass index; *IHD* ischaemic heart disease; *IQR* interquartile range; *MAP* mean arterial pressure; *HR* heart rate; *T2DM* type 2 diabetes mellitus; *eGFR* estimated glomerular filtration rate; *HbA1c* glycated haemoglobin A1c; *LDL* low-density lipoprotein; *HDL* high-density lipoprotein; *UACR* urine albumin-to-creatinine ratio; *ACE* angiotensin converting enzyme; *ARB* aldosterone receptor blocker; *SGLT-2 inhibitor* sodium-glucose cotransporter-2 inhibitor; *DPP-4* dipeptidyl peptidase 4 inhibitor; *GLP-1 agonists* glucagon-like peptide-1 agonists

*Comparison of medians between patients with and without microvascular complications

**Table 2:** Overview of subgroups of microvascular complications in 222 patients

	No microvascular complications (n = 111)	1 microvascular complication (n = 75)	2–3 microvascular complications (n = 36)
Albuminuria*			
No albuminuria	111 (100)	47 (63)	12 (33)
Microalbuminuria	0 (0)	25 (33)	23 (64)
Macroalbuminuria	0 (0)	3 (4)	1 (3)
Nephropathy			
No CKD	7 (6)	2 (3)	3 (8)
Mild CKD	96 (86)	65 (87)	29 (81)
Moderate CKD (stage IIIa)	8 (7)	8 (11)	4 (11)
Diabetic retinopathy*			
No retinopathy	104 (94)	45 (60)	4 (11)
Mild NPDR	0 (0)	15 (20)	11 (30.5)
Moderate NPDR	0 (0)	8 (11)	11 (30.5)
Severe NPDR	0 (0)	2 (3)	5 (14)
PDR	0 (0)	4 (5)	5 (14)
Peripheral neuropathy*			
Normal sensitivity	87 (78)	48 (64)	9 (25)
Reduced sensitivity	0 (0)	17 (23)	24 (67)
No sensitivity	0 (0)	1 (1)	3 (8)

*CKD* chronic kidney disease; *NPDR* non-proliferative diabetic retinopathy; *PDR* proliferative diabetic retinopathy

*Only albuminuria, diabetic retinopathy and peripheral neuropathy were considered as microvascular complications

### Characteristics stratified by diabetes-related microvascular complications

Only minor differences in characteristics were observed between patients without microvascular complications and patients with any number of microvascular complications (Table 1). Among the patients with microvascular complications, a higher proportion were men (76% vs 60%). Patients with complications had a longer history of diabetes (16 ± 11 years vs 11 ± 9 years) and a higher HR (71 ± 12 beats/minute vs 67 ± 13 beats/minute). Furthermore, patients with complications had a lower eGFR (72 ± 12 ml/min/m^2^ vs 75 ± 9 ml/min/m^2^) and higher levels of HbA1c (63 ± 15 mmol/mol vs 59 ± 13 mmol/mol) than patients with no complications. There were significantly higher median values of TnT and UACR in patients with complications and a higher proportion of patients using ASAs (14% vs 5%) and calcium blockers (29% vs 17%) in the group with complications than in the group with no complications. Interestingly, there was no difference in the proportions of patients using statins (69% vs 62%).

**Fig. 3:**
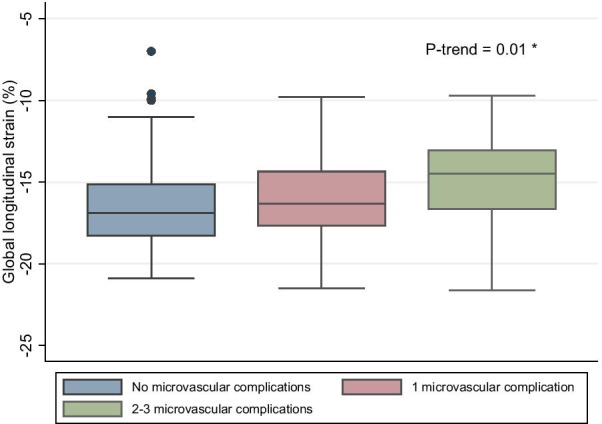
Box plot of global longitudinal strain stratified by diabetes-related microvascular complications in 222 patients. Boxplot with median global longitudinal strain and corresponding interquartile range. The whiskers represent the variability outside the interquartile range and dots outside the whiskers are statistical outliers.*Cuzick’s test for trend across groups

There was a difference in GLS (− 16.4 ± 2.5% vs − 16.0 ± 2.5% vs − 14.9 ± 2.8%, *p* = 0.01) among patients with no complications, 1 complication and 2–3 complications, respectively (Table 3). A trend was observed with an increasing burden of microvascular complications and reduced global longitudinal strain (Fig. 3) (*p* = 0.01). There was also a difference in e’ (0.09 ± 0.02 cm/sec vs 0.09 ± 0.05 cm/sec vs 0.08 ± 0.02 cm/s, *p* = 0.03) among patients with no complications, 1 complication and 2–3 complications.

**Table 3:** Characteristics of echocardiographic and CCTA parameters stratified by microvascular complications in 222 patients

	No microvascular complications (n = 111)	1 microvascular complications (n = 75)	2–3 microvascular complications (n = 36)	*p*
Echocardiography				
LVEF (%)	56 ± 5	54 ± 6	55 ± 5	ns
GLS (%)	− 16.4 ± 2.5	− 16.0 ± 2.5	− 14.9 ± 2.8	0.01
LAVI (ml/m^2^)	22 ± 7	23 ± 7	21 ± 5	ns
LVMI (g/m^2^)	71 ± 23	77 ± 21	71 ± 19	ns
e’ (cm/sec)	0.09 ± 0.02	0.09 ± 0.05	0.08 ± 0.02	0.03
IVRT (msec)	82 ± 20	84 ± 17	83 ± 17	ns
E/e’	9.2 ± 2.9	9.7 ± 3.1	10.0 ± 2.7	ns
E/A	1.1 ± 0.3	1.0 ± 0.3	0.9 ± 0.3	ns
DCT (msec)	221 ± 56	221 ± 53	209 ± 44	ns
Computed tomography				
CAC [IQR]	39 [0–239]	22 [0–180]	130 [30–720]	ns*
PB (%)	20 ± 17	22 ± 16	27 ± 18	ns
PB LAD/LM (%)	36 ± 11	35 ± 9	40 ± 11	ns
PB RCA (%)	26 ± 8	27 ± 10	30 ± 11	ns
PB CX (%)	33 ± 12	31 ± 11	37 ± 13	ns

*CCTA* coronary computed tomography angiography; *ns* not significant; *LVEF* left ventricular ejection fraction; *GLS* global longitudinal strain; *LAVI* left atrial volume indexed; *LVMI* left ventricular mass indexed; *e’* tissue Doppler average early diastolic mitral annulus velocity; *IVRT* tissue Doppler average isovolumetric relaxation time; *E/é* ratio of transmitral Doppler early peak velocity (E) and tissue Doppler average early diastolic mitral annulus velocity (e'); *E/A* ratio of transmitral Doppler early peak velocity (E) and transmitral Doppler peak late velocity (A); *DCT* deceleration time; *CAC* coronary artery calcium; *IQR* interquartile range; *PB* plaque burden; *LAD/LM* left anterior descending coronary artery/left main; *RCA* right coronary artery; *CX* circumflex artery

*Comparison of medians between patients with and without microvascular complications

No difference in CAC or PB was found among patients with no complications, 1 complication and 2–3 complications (Table 3). Furthermore, there were no differences in LVEF, LAVI, LVMI, IVRT, E/e’, E/A or DCT between the groups.

### Coronary artery plaque burden

**Fig. 4:**
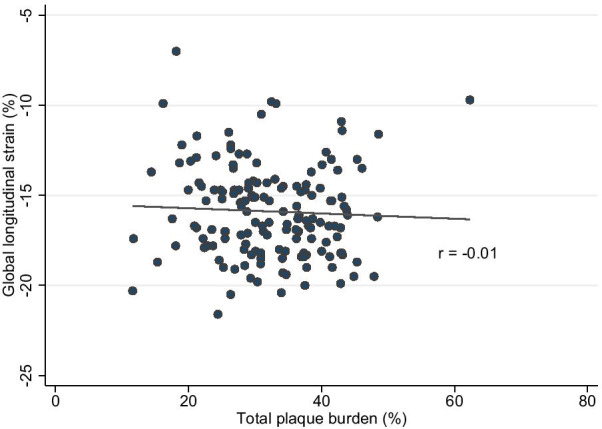
Scatter plot of global longitudinal strain and total plaque burden in 152 patients with plaque volumes > 0 mm^3^. Two-way scatterplot with dots representing total plaque burden plotted against global longitudinal strain with a regression line (r represents the slope of the best linear fit)

An increase in the number of microvascular complications (β 2.97 [95% CI 0.42–5.51], *p* = 0.02) was associated with increasing PB in a univariable exploratory regression model. A negligible correlation was observed between PB and GLS (Fig. 4), nonetheless a one-unit increase in PB improved GLS by 0.01 percentage point. Overall, there was no linear relationship between PB and GLS.

One hundred and fifty-two (68%) patients had plaque volumes > 0 mm^3^. The majority of plaques were located in the LAD/LM with a median TPV of 154 mm^3^ (IQR [0–352]), whereas the median TPV was 0 mm^3^ (IQR [0–273]) in the RCA and 0 mm^3^ (IQR [0–177]) in the CX.

### Linear regression models

In the univariable linear regression model, patients with T2DM (β 1.30 [95% CI 0.50–2.11]) had significantly reduced GLS compared to patients with type 1 diabetes (T1D) (Table 4). Men (β 0.60 [95% CI − 0.13–1.34]) did not have significantly reduced GLS compared to women. An increasing number of microvascular complications (β 0.71 [95% CI 0.59–0.82]) was associated with reduced GLS. A one unit increase in BMI (β 0.14 [95% CI 0.08–0.21]) and MAP (β 0.07 [95% CI 0.04–0.10]) were associated with reduced GLS. Additionally, a one unit increase in e’ (β − 19.8 [95% CI − 30.2–9.42]) was associated with improved GLS. A one unit increase in PB was not associated with GLS (β 0.01 [95% CI − 0.01–0.03]). The use of acetylsalicylic acid or calcium blockers did not have a significant impact on GLS.

**Table 4:** Uni- and multivariable linear regression models of global longitudinal strain in 222 patients

Clinical	Univariable				Multivariable*			
	β	95% CI		*p*	β	95% CI		*p*
Age (years)	0.01	− 0.03	0.04	ns				
Men	0.60	− 0.13	1.34	ns				
BMI (kg/m^2^)	0.14	0.08	0.21	< 0.001	0.12	0.05	0.19	0.01
MAP (mmHg)	0.07	0.04	0.10	< 0.001	0.05	0.01	0.08	0.01
Diabetes duration (years)	− 0.03	− 0.06	0.01	ns				
Microvascular complications	0.71	0.59	0.82	< 0.001	0.50	0.11	0.88	0.01
Biochemistry								
HbA1c (mmol/mol)	0.01	− 0.01	0.04	ns				
Medication								
Acetylsalicylic acid	0.06	− 1.09	1.22	ns				
Calcium blockers	0.02	− 0.79	0.84	ns				
Type of diabetes								
T2DM	1.30	0.50	2.11	0.01	0.56	− 0.26	1.39	ns
Echocardiography								
e’ (cm/sec)	− 19.8	− 30.2	− 9.42	< 0.001	− 12.7	− 22.8	− 2.54	0.02
Computed tomography								
PB (%)	0.01	− 0.01	0.03	ns				

*β* beta coefficient; *95% CI* 95% confidence interval; *ns* not significant; *BMI* body mass index; *MAP* mean arterial pressure; *HbA1c* glycated haemoglobin A1c; *T2DM* type 2 diabetes mellitus; *e’* tissue Doppler average early diastolic mitral annulus velocity; *PB* plaque burden

*Data were available for 213 patients

In the multivariable linear regression model, T2DM (β 0.56 [95% CI − 0.26–1.39]) did not remain significantly associated with reduced GLS compared to patients with T1D. Nevertheless, the increase in the number of microvascular complications (β 0.50 [95% CI 0.11–0.88]) remained significantly associated with reduced GLS. Finally, a one unit increase in BMI (β 0.12 [95% CI 0.05–0.19]) and MAP (β 0.05 [95% CI 0.01–0.08]) remained significantly associated with reduced GLS, whereas a one unit increase in e’ (β − 12.7 [95% CI − 22.8–2.54]) remained significantly associated with improved GLS (Table 4). These findings were consistent in an additional multivariable regression model with concomitant adjustment for HR and LVM with the exception of e’. MAP and LVM were not correlated (r = 0.02). Finally, the findings remained unchanged, when previously excluded patients with significant CAD were added to the multivariable regression model (Additional file 1).

#### Associations between subgroups of microvascular complications and GLS

Presence of albuminuria was associated with significantly reduced GLS (β 1.10 [95% CI 0.30–1.90], *p* = 0.01). Subgroup analysis of microalbuminuria was associated with significantly reduced GLS (β 1.00 [95% CI 0.18–1.83], *p* = 0.02) compared to normoalbuminuria, whereas macroalbuminuria was not associated with reduced GLS (β 2.26 [95% CI − 0.30–4.82], *p* = 0.08).

None of the subgroups of retinopathies or neuropathies were associated with GLS. In addition, presence of retinopathy was not associated with GLS compared to no retinopathy (*p* = 0.22) and likewise the presence of neuropathy was not associated with GLS compared to no neuropathy (*p* = 0.15).

In a multivariable exploratory regression model, the presence of albuminuria was associated with significantly reduced GLS (β 1.03 [95% CI 0.15–1.92], *p* = 0.02) compared to no albuminuria, whereas the presence of neuropathy (β 0.54 [95% CI − 0.40–1.48]) and retinopathy (β 0.10 [95% CI − 0.79–0.98]) were not associated with significantly reduced GLS.

### Reproducibility of GLS and PB

Data on GLS showed an intra-variability value of 0.42 [− 1.75–2.59] and inter-variability value of − 0.22 [− 2.75–2.30], and the corresponding correlation coefficients were 0.81 and 0.75, respectively. Data on PB showed an intra-variability value of 0.21 [− 4.90–5.32] and inter-variability value of 0.92 [− 6.06–7.89], and the corresponding correlation coefficients were 0.98 and 0.96, respectively.

## Discussion

This is the first study to assess the association between diabetes-related microvascular complications, GLS, and PB in asymptomatic patients with DM and non-obstructive CAD. The novel findings of this study are as follows: 1) an association between an increased number of diabetes-related microvascular complications and an unfavorable impact on GLS was observed, independent of other known cardiovascular risk factors; 2) plaque burden was not associated with GLS; and 3) diabetes-related microvascular complications were associated with plaque burden.

### Diabetes-related microvascular complications

In this study, an association between an increased number of microvascular complications and reduced GLS was observed. Previous studies have investigated the impact of an increasing number of microvascular complications and GLS and detected a significant association. Of the microvascular complications, albuminuria has been investigated extensively in patients with DM [22, 23], and albuminuria was associated with reduced LV systolic function, including GLS. Retinopathy [24] and peripheral neuropathy [25] have also been investigated and are also observed to occur with reduced LV systolic function.

The majority of patients in this study had either no or 1 microvascular complication and did not differ significantly in GLS. Patients with either 2 or 3 microvascular complications accounted for a relatively small proportion and did not differ significantly in GLS. The incremental effect of microvascular complications remained an independent predictor of reduced GLS in the multivariable linear regression model. The severity of the microvascular complications was considered and did not show any significant associations with GLS in the explanatory regression models, except for patients with microalbuminuria. Only a small fraction of patients had macroalbuminuria, which could be responsible for the lack of an observed association between macroalbuminuria and reduced GLS. Our findings resemble those of a study by Jensen et al. [11], which investigated myocardial dysfunction in relation to albuminuria in 1065 patients with T1D and 198 healthy control subjects. Jensen et al. observed that GLS was reduced in patients with T1D compared to healthy control subjects, and these findings were primarily driven by albuminuria. These patients were asymptomatic, but there was no evaluation of subclinical CAD. In addition, a study by Jørgensen et al. [12] showed that 915 patients with T2DM with microalbuminuria had diastolic dysfunction, whereas patients with macroalbuminuria had reduced systolic function. These patients were asymptomatic, but there was no evaluation of subclinical CAD. Our results, along with the findings by Jensen et al. and Jørgensen et al., indicate that an increasing burden of microvascular complications seems to have a negative impact on GLS regardless of CAD in asymptomatic patients with DM. Furthermore, microvascular complications such as albuminuria could represent a marker of vascular damage and endothelial dysfunction.

Endothelial dysfunction including coronary microvascular dysfunction (CMD) has been investigated and previous studies have observed an association between CMD and GLS in symptomatic patients [13, 26, 27]. CMD was evaluated by coronary flow velocity reserve (CFVR), whereas a CFVR < 2 was considered as CMD. Michelsen et al. [26] investigated the association between CMD, LV diastolic function and LV systolic function in 963 women with no significant coronary artery stenosis (< 50%) by invasive coronary angiography. Michelsen et al. observed that CMD was associated with increased age and a higher resting HR. They also observed that GLS reserve was significantly lower in women with reduced CMD, whereas there were no significant changes in diastolic function. CMD was not assessed in our study, but an increasing burden of microvascular complications could be a surrogate for endothelial dysfunction such as CMD; however, further studies are warranted to better understand these mechanisms.

A study by Mochizuki et al. [18] investigated the association between microvascular complications and GLS in 144 asymptomatic patients with DM and preserved LVEF (≥ 50%). Mochizuki et al. observed that microvascular complications, hypertriglyceridaemia and overweight were closely associated with reduced GLS. The patients recruited for the study all underwent treadmill exercise or stress myocardial perfusion scintigraphy, and were included only when no ischaemic response was detected. The patients were presented as asymptomatic patients but were recruited for the study during a hospital admission, whereas the cause of admission was not specified. Additionally, there was no specific evaluation of CAD, although a functional test for ischaemia was performed before inclusion. Despite these differences in study design, our results were in accordance with those of Mochizuki et al.

### Cardiovascular risk factors

A study by Ballo et al. [28] investigated the relationship between LV systolic function, hypertension and DM in patients without angina. They observed an additive effect of DM itself on hypertension in relation to LV systolic function. Ballo et al. did not observe hypertension to be associated with reduced LV systolic function and discussed a potential mechanism through biochemical pathways (calcium regulation and insulin signaling). Our findings showed that MAP remained a significant predictor of GLS in the multivariable regression model, which could explain hypertension-induced nephropathy in patients with DM over time.

Furthermore, a study by Ng et al. [29] investigated 337 patients with T2DM and 316 patients without DM in relation to the impact of BMI and myocardial systolic and diastolic function in patients without known or suspected CAD. The authors observed that both diabetes and increased BMI were associated with reduced myocardial systolic and diastolic function and that increased BMI was associated with greater LV myocardial dysfunction than T2DM. Ng et al. discussed obesity-related metabolic, inflammatory and neurohormonal changes. Our findings showed that BMI remained a significant predictor of GLS in the multivariable regression model, which supports the findings by Ng et al. However, we did not evaluate the impact of BMI in terms of metabolic syndrome and potential inflammatory causes of reduced GLS.

A study by Garofolo et al. [30] investigated an association between microvascular complications and an increased risk of major cardiovascular outcomes and all-cause mortality in 774 patients with T1D over 10 years of follow-up. They stated that the presence and number of microvascular complications should be considered in the risk stratification of patients, which is in accordance with our current findings.

### Coronary artery plaque burden

There was a negligible correlation between PB and GLS, and PB was not associated with GLS in the univariable regression model. There was no assessment of contractile reserve, but only an assessment of systolic function during rest, which could also explain a lack of correlation between PB and GLS. No systemic bias was suspected on the basis of the reproducibility of PB in this study.

Previously published studies involving asymptomatic patients with DM have mainly focused on CAC and not PB, why potential significant and flow limiting CAD was not excluded. A study by Scholte et al. [8] showed that patients with T2DM with coronary atherosclerosis exhibited reduced GLS compared to patients with T2DM and no coronary atherosclerosis. Another study by Venkataraman et al. [31] investigated the association among CAC, conventional cardiovascular risk factors and subclinical left ventricular dysfunction in patients with a low-intermediate risk of CAD. They concluded that atherosclerosis was not associated with subclinical LV dysfunction, and the findings indicated no linear relationship in a low-risk group. The findings by Venkataraman et al. could support a selection bias of patients in this study and explain a lack of association between PB and LV systolic function.

There was a significant association between an increasing number of microvascular complications and increasing PB in the univariable explanatory regression model. A study by Lovshin [32] et al. investigated the relationships between atherosclerotic burden and neuropathy, retinopathy and diabetic kidney disease in 69 patients with T1D compared to 73 matched control subjects. They observed that the presence of microvascular complications such as neuropathy and retinopathy was associated with more severe atherosclerosis evaluated by CAC. Our findings indicate that microvascular complications could contribute to the development of PB in terms of calcified plaques.

### Mechanisms for impairment of left ventricular function

The exact mechanism for diabetes-related microvascular complications, specifically albuminuria and the development of cardiovascular disease, is not yet fully understood.

A study by *Potier* et al. investigated 175 patients with and without DM to evaluate the relationship between DM, CMD and microvascular complications in patients without known cardiovascular disease (CVD). They underwent a rubidium positron emission tomography (Rb-PET) for screening of CAD, and a myocardial blood flow reserve (MFR) was measured. Potier et al. observed that patients with DM had a higher prevalence of reduced CMD than patients without DM. Furthermore, they detected a clear reduction in MFR in the patients with the highest level of albuminuria, which made the authors hypothesize a common pathway for both CMD and albuminuria. One mechanism suggested by Potier et al. [33] is that DM eventually leads to microvascular complications, which ultimately lead to end-organ damage, including LV systolic dysfunction secondary to reduced myocardial perfusion [33]. Our findings showed that the impact of microvascular complications on GLS was mainly driven by the presence of albuminuria, which could support the mechanism mentioned by Potier et al.

Another mechanism was suggested by *Kawata* et al. [34]*.* They investigated the relationship between CFR and diastolic function in 67 asymptomatic patients with T2DM and 14 controls. They observed an inverse relationship between CFR and left ventricular filling pressure, but they also detected lower values of CFR in patients with T2DM than in controls. Kawata et al. did not suggest any causality between increased left ventricular filling pressure and reduced CFR, but proposed a possible mechanism involving oxidative stress [35].

### Clinical implications

An incremental effect of microvascular complications on GLS was observed in asymptomatic patients with DM and no significant CAD. Even asymptomatic patients with DM and no microvascular complications seem to have a reduced GLS compared to the background population. Subtle changes in LV systolic function were detected with advanced echocardiography and not with conventional LVEF. Our findings emphasize the need for an echocardiography on a low indication.

### Study limitations

One limitation in this study may be the selection of patients due to missing data on GLS. Second, this population consisted of patients with both T1D and T2DM, of which the majority had T2DM. The findings in this study could potentially be driven by patients with T2DM, despite suitable adjustment in multivariable regression models. Third, a larger study is recommended in order to increase the statistical power, which could be achieved by including a more balanced distribution of subgroups of microvascular complications, proportions of patients with 2–3 diabetes-related microvascular complications and type of diabetes. Fourth, the specific threshold value of significant coronary artery stenosis was established by the author group and could represent a selection bias, though a post-hoc analysis showed that our findings remained unchanged with the addition of previously excluded patients due to significant CAD. Fifth and finally, there was a significantly lower proportion of women and data was not obtained for gender-specific comorbidity related to cardiovascular disease.

## Conclusions

An increased burden of diabetes-related microvascular complications was independently associated with reduced GLS in asymptomatic patients with DM and non-obstructive coronary arteries. Finally, the burden of microvascular complications was associated with increasing PB, whereas PB was not associated with GLS.

## Supplementary Information

**Additional file 1**. Multivariable linear regression model of global longitudinal strain in patients with and without significant coronary artery disease.

## Data Availability

The datasets used and/or analysed during the current study are available from the corresponding author on reasonable request.

## Abbreviations

A: Transmitral Doppler late peak velocity
BMI: Body mass index
BSA: Body surface area
DM: Diabetes mellitus
E: Transmitral Doppler early peak velocity
e’: Tissue Doppler average early diastolic mitral annulus velocity
eGFR: Estimated glomerular filtration rate
GLS: Global longitudinal strain
HbA1c: Glycated haemoglobin A1c
IVRT: Isovolumetric relaxation time
MAP: Mean arterial pressure
LAVI: Left atrial volume index
LVEF: Left ventricular ejection fraction
LVMI: Left ventricular mass index
PB: Plaque burden
T1D: Type 1 diabetes
T2DM: Type 2 diabetes mellitus

## References

[1] Pan W, Lu H, Lian B, Liao P, Guo L, Zhang M (2019). Prognostic value of HbA1c for in-hospital and short-term mortality in patients with acute coronary syndrome: a systematic review and meta-analysis. Cardiovasc Diabetol.

[2] Marso SP, Daniels GH, Brown-Frandsen K, Kristensen P, Mann JF, Nauck MA (2016). Liraglutide and cardiovascular outcomes in type 2 diabetes. N Engl J Med.

[3] Perkovic V, Jardine MJ, Neal B, Bompoint S, Heerspink HJL, Charytan DM (2019). Canagliflozin and renal outcomes in type 2 diabetes and nephropathy. N Engl J Med.

[4] Nathan DM, Genuth S, Lachin J, Cleary P, Crofford O, Davis M (1993). The effect of intensive treatment of diabetes on the development and progression of long-term complications in insulin-dependent diabetes mellitus. N Engl J Med.

[5] Stratton IM, Adler AI, Neil HA, Matthews DR, Manley SE, Cull CA (2000). Association of glycaemia with macrovascular and microvascular complications of type 2 diabetes (UKPDS 35): prospective observational study. BMJ.

[6] Davis TM, Coleman RL, Holman RR (2013). Prognostic significance of silent myocardial infarction in newly diagnosed type 2 diabetes mellitus: United Kingdom Prospective Diabetes Study (UKPDS) 79. Circulation.

[7] Kawano Y, Takemoto M, Mito T, Morisaki H, Tanaka A, Sakaki Y (2016). Silent myocardial ischemia in asymptomatic patients with type 2 diabetes mellitus without previous histories of cardiovascular disease. Int J Cardiol.

[8] Scholte AJ, Nucifora G, Delgado V, Djaberi R, Boogers MJ, Schuijf JD (2011). Subclinical left ventricular dysfunction and coronary atherosclerosis in asymptomatic patients with type 2 diabetes. Eur J Echocardiogr.

[9] Roos CJ, Scholte AJ, Kharagjitsingh AV, Bax JJ, Delgado V (2014). Changes in multidirectional LV strain in asymptomatic patients with type 2 diabetes mellitus: a 2-year follow-up study. Eur Heart J Cardiovasc Imaging.

[10] Ernande L, Bergerot C, Girerd N, Thibault H, Davidsen ES, Gautier Pignon-Blanc P (2014). Longitudinal myocardial strain alteration is associated with left ventricular remodeling in asymptomatic patients with type 2 diabetes mellitus. J Am Soc Echocardiogr.

[11] Jensen MT, Sogaard P, Andersen HU, Bech J, Fritz Hansen T, Biering-Sørensen T (2015). Global longitudinal strain is not impaired in type 1 diabetes patients without albuminuria: the Thousand & 1 study. JACC Cardiovasc Imaging.

[12] Jørgensen PG, Biering-Sørensen T, Mogelvang R, Fritz-Hansen T, Vilsbøll T, Rossing P (2018). Presence of micro- and macroalbuminuria and the association with cardiac mechanics in patients with type 2 diabetes. Eur Heart J Cardiovasc Imaging.

[13] Løgstrup BB, Høfsten DE, Christophersen TB, Møller JE, Bøtker HE, Pellikka PA (2012). Correlation between left ventricular global and regional longitudinal systolic strain and impaired microcirculation in patients with acute myocardial infarction. Echocardiography.

[14] Akasaka T, Yoshida K, Hozumi T, Takagi T, Kaji S, Kawamoto T (1997). Retinopathy identifies marked restriction of coronary flow reserve in patients with diabetes mellitus. J Am Coll Cardiol.

[15] Alberti KG, Zimmet PZ (1998). Definition, diagnosis and classification of diabetes mellitus and its complications. Part 1: diagnosis and classification of diabetes mellitus provisional report of a WHO consultation. Diabet Med.

[16] Adelborg K, Szentkúti P, Henriksen JE, Thomsen RW, Pedersen L, Sundbøll J (2020). Cohort profile: the Funen Diabetes Database—a population-based cohort of patients with diabetes in Denmark. BMJ Open.

[17] Cerqueira MD, Weissman NJ, Dilsizian V, Jacobs AK, Kaul S, Laskey WK (2002). Standardized myocardial segmentation and nomenclature for tomographic imaging of the heart. A statement for healthcare professionals from the Cardiac Imaging Committee of the Council on Clinical Cardiology of the American Heart Association. Circulation.

[18] Mochizuki Y, Tanaka H, Matsumoto K, Sano H, Toki H, Shimoura H (2015). Clinical features of subclinical left ventricular systolic dysfunction in patients with diabetes mellitus. Cardiovasc Diabetol.

[19] Enomoto M, Ishizu T, Seo Y, Kameda Y, Suzuki H, Shimano H (2016). Myocardial dysfunction identified by three-dimensional speckle tracking echocardiography in type 2 diabetes patients relates to complications of microangiopathy. J Cardiol.

[20] Roy S, Kant R, Kumar B, Khapre M, Bairwa M (2020). Systolic dysfunction in asymptomatic type 2 diabetic patients, a harbinger of microvascular complications: a cross-sectional study from North India. Diab Vasc Dis Res.

[21] Austin PC, Steyerberg EW (2015). The number of subjects per variable required in linear regression analyses. J Clin Epidemiol.

[22] Hanna DB, Xu S, Melamed ML, Gonzalez F, Allison MA, Bilsker MS (2017). Association of albuminuria with cardiac dysfunction in US Hispanics/Latinos. Am J Cardiol.

[23] Katz DH, Burns JA, Aguilar FG, Beussink L, Shah SJ (2014). Albuminuria is independently associated with cardiac remodeling, abnormal right and left ventricular function, and worse outcomes in heart failure with preserved ejection fraction. JACC Heart Fail.

[24] Nouhravesh N, Andersen HU, Jensen JS, Rossing P, Jensen MT (2016). Retinopathy is associated with impaired myocardial function assessed by advanced echocardiography in type 1 diabetes patients—The Thousand & 1 Study. Diabetes Res Clin Pract.

[25] Mochizuki Y, Tanaka H, Matsumoto K, Sano H, Toki H, Shimoura H (2015). Association of peripheral nerve conduction in diabetic neuropathy with subclinical left ventricular systolic dysfunction. Cardiovasc Diabetol.

[26] Michelsen MM, Pena A, Mygind ND, Bech J, Gustafsson I, Kastrup J (2018). Coronary microvascular dysfunction and myocardial contractile reserve in women with angina and no obstructive coronary artery disease. Echocardiography.

[27] Lowenstein JA, Caniggia C, Rousse G, Amor M, Sánchez ME, Alasia D (2014). Coronary flow velocity reserve during pharmacologic stress echocardiography with normal contractility adds important prognostic value in diabetic and nondiabetic patients. J Am Soc Echocardiogr.

[28] Ballo P, Cameli M, Mondillo S, Giacomin E, Lisi M, Padeletti M (2010). Impact of diabetes and hypertension on left ventricular longitudinal systolic function. Diabetes Res Clin Pract.

[29] Ng ACT, Prevedello F, Dolci G, Roos CJ, Djaberi R, Bertini M (2018). Impact of diabetes and increasing body mass index category on left ventricular systolic and diastolic function. J Am Soc Echocardiogr.

[30] Garofolo M, Gualdani E, Giannarelli R, Aragona M, Campi F, Lucchesi D (2019). Microvascular complications burden (nephropathy, retinopathy and peripheral polyneuropathy) affects risk of major vascular events and all-cause mortality in type 1 diabetes: a 10-year follow-up study. Cardiovasc Diabetol.

[31] Venkataraman P, Wright L, Huynh Q, Marwick TH (2020). Independence of coronary artery disease to subclinical left ventricular dysfunction. Echocardiography.

[32] Lovshin JA, Bjornstad P, Lovblom LE, Bai JW, Lytvyn Y, Boulet G (2018). Atherosclerosis and microvascular complications: results from the Canadian study of longevity in type 1 diabetes. Diabetes Care.

[33] Potier L, Chequer R, Roussel R, Mohammedi K, Sismail S, Hartemann A (2018). Relationship between cardiac microvascular dysfunction measured with 82Rubidium-PET and albuminuria in patients with diabetes mellitus. Cardiovasc Diabetol.

[34] Kawata T, Daimon M, Miyazaki S, Ichikawa R, Maruyama M, Chiang SJ (2015). Coronary microvascular function is independently associated with left ventricular filling pressure in patients with type 2 diabetes mellitus. Cardiovasc Diabetol.

[35] Mourmoura E, Vial G, Laillet B, Rigaudière JP, Hininger-Favier I, Dubouchaud H (2013). Preserved endothelium-dependent dilatation of the coronary microvasculature at the early phase of diabetes mellitus despite the increased oxidative stress and depressed cardiac mechanical function ex vivo. Cardiovasc Diabetol.

