# Supplementary material for: Diabetic microvascular complications are associated with reduced global longitudinal strain independent of atherosclerotic coronary artery disease in asymptomatic patients with diabetes mellitus: a cross-sectional study
Source: BMC Cardiovasc Disord. 2021 Jun 2;21:269. doi: 10.1186/s12872-021-02063-w (PMC8173786; doi:10.1186/s12872-021-02063-w)
Supplement: Supplementary file 1 — Additional file 1. Multivariable linear regression model of global longitudinal strain in patients with and without significant coronary artery disease. [file 12872_2021_2063_MOESM1_ESM.pdf]

Additional file 1

Multivariable linear regression model of global longitudinal strain in patients with and without significant coronary artery disease *

	Multivariable **			
	β	95% CI		P
**Clinical**				
Age (years)				
Men				
BMI (kg/m²)	0.13	0.06	0.19	< 0.001
MAP (mmHg)	0.04	0.01	0.07	0.01
Diabetes duration (years)				
Microvascular complications	0.50	0.14	0.86	0.01
**Biochemistry**				
HbA1c (mmol/mol)				
**Medication**				
Acetylsalicylic acid				
Calcium blockers				
**Type of diabetes**				
T2DM	0.69	-0.11	1.49	ns
**Echocardiography**				
e' (cm/sec)	-12.42	-22.34	-2.49	0.01
**Computed tomography**				
PB (%)				

β, beta coefficient; 95% CI, 95% confidence interval; ns, not significant; BMI, body mass index; MAP, mean arterial pressure; HbA1c, glycated haemoglobin A1c; T2DM, type 2 diabetes mellitus; e', tissue Doppler mean early diastolic mitral annulus velocity; PB, plaque burden.

* All patients had a PB < 70%

** Data were available for 244 patients.
